# Potassium [1-(*tert*-but­oxy­carbon­yl)-1*H*-indol-3-yl]trifluoro­borate hemihydrate

**DOI:** 10.1107/S1600536812014225

**Published:** 2012-04-06

**Authors:** Guillaume Berionni, Peter Mayer, Herbert Mayr

**Affiliations:** a Ludwig-Maximilians-Universität, Department of Chemistry, Butenandtstrasse 5–13, 81377 München, Germany

## Abstract

The asymmetric unit of the title salt, K^+^·C_13_H_14_BF_3_NO_2_·0.5H_2_O, consists of two derivatized indolyltrifluoridoborate anions, two potassium cations and one water mol­ecule. Within the indolyltrifluoro­borate anions, the least-square planes consisting of the carboxyl group and the adjacent quarternary C atom of the *tert*-butyl groups deviate significantly from coplanarity with the indolyl planes [20.44 (11) and 21.02 (10)°]. The potassium ions are coordinated by six atoms (one K^+^ ion by two O and four F atoms, and the second K^+^ ion by one O and five F atoms), however, one of the potassium ions undergoes an additional weak potassium–π inter­action (K⋯centroid = 3.722 Å). The packing is stabilized by sequential O—H⋯O hydrogen bonds along [100] between water mol­ecules and also by O—H⋯F hydrogen bonds.

## Related literature

For background to organotrifluoro­borates and the synthesis, see: Mothes *et al.* (2008 ▶); Molander *et al.* (2009 ▶); Kassis *et al.* (2009 ▶); Reiter *et al.* (2010 ▶); Darses & Genet (2008 ▶). For related structures, see: Baran *et al.* (2005 ▶); Davies *et al.* (2005 ▶, 2007 ▶); Lu & Lin (2011 ▶).
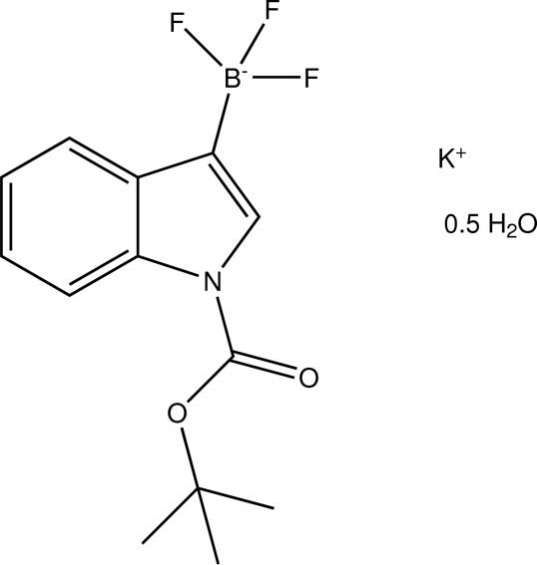


## Experimental

### 

#### Crystal data

K^+^·C_13_H_14_BF_3_NO_2_·0.5H_2_O
*M* *_r_* = 332.17
Orthorhombic, 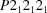

*a* = 5.8428 (1) Å
*b* = 16.3177 (2) Å
*c* = 32.1286 (5) Å
*V* = 3063.17 (8) Å^3^
*Z* = 8
Mo *K*α radiation
μ = 0.38 mm^−1^
*T* = 173 K
0.27 × 0.19 × 0.10 mm

#### Data collection

Nonius KappaCCD diffractometer
24771 measured reflections
7001 independent reflections
6369 reflections with *I* > 2σ(*I*)
*R*_int_ = 0.027

#### Refinement

*R*[*F* ^2^ > 2σ(*F* ^2^)] = 0.032
*wR*(*F* ^2^) = 0.075
*S* = 1.03
7001 reflections
400 parameters
2 restraints
H atoms treated by a mixture of independent and constrained refinement
Δρ_max_ = 0.30 e Å^−3^
Δρ_min_ = −0.24 e Å^−3^
Absolute structure: Flack (1983 ▶), 2995 Friedel pairs
Flack parameter: 0.00 (3)

### 

Data collection: *COLLECT* (Hooft, 2004 ▶); cell refinement: *SCALEPACK* (Otwinowski & Minor, 1997 ▶); data reduction: *DENZO* (Otwinowski & Minor, 1997 ▶) and *SCALEPACK*; program(s) used to solve structure: *SIR97* (Altomare *et al.*, 1999 ▶); program(s) used to refine structure: *SHELXL97* (Sheldrick, 2008 ▶); molecular graphics: *ORTEP-3* (Farrugia, 1997 ▶); software used to prepare material for publication: *PLATON* (Spek, 2009 ▶).

## Supplementary Material

Crystal structure: contains datablock(s) I, global. DOI: 10.1107/S1600536812014225/hp2034sup1.cif

Structure factors: contains datablock(s) I. DOI: 10.1107/S1600536812014225/hp2034Isup2.hkl

Additional supplementary materials:  crystallographic information; 3D view; checkCIF report

## Figures and Tables

**Table 1:** Hydrogen-bond geometry (Å, °)

*D*—H⋯*A*	*D*—H	H⋯*A*	*D*⋯*A*	*D*—H⋯*A*
O5—H51⋯F3^i^	0.81 (1)	2.14 (1)	2.903 (2)	156 (3)
O5—H51⋯F1^i^	0.81 (1)	2.62 (2)	3.188 (2)	128 (2)
O5—H52⋯O5^ii^	0.81 (1)	2.41 (1)	3.1968 (16)	164 (3)

Symmetry codes: (i) 
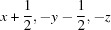
; (ii) 
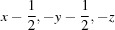
.

**Fig. 1.**
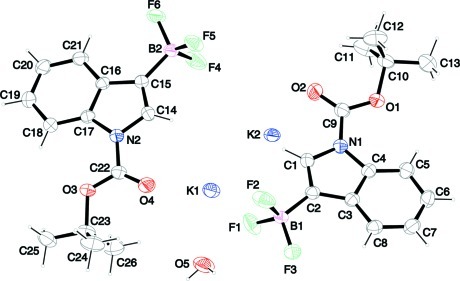
The molecular structures of the asymmetric unit (contains two formula units of the title compound), with atom labels and anisotropic displacement ellipsoids (drawn at 50% probability level) for non-H atoms.

**Fig. 2.**
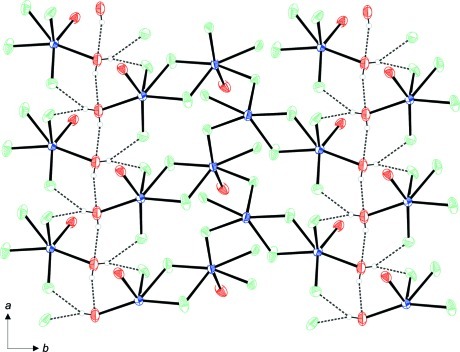
View on a layer formed by potassium ions and their coordinating O and F atoms. All other atoms have been omitted for clarity. Dashed lines indicate hydrogen bonds, fat solid lines K–O and K–F bonds.

**Fig. 3.**
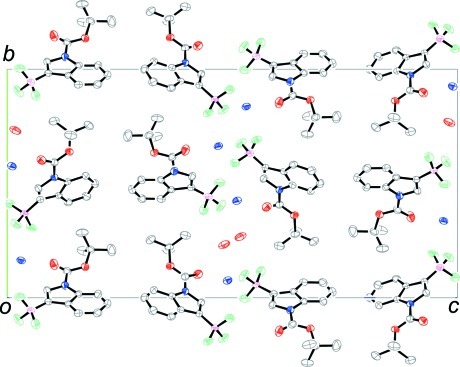
The packing of the title compound viewed along [100]. Hydrogen atoms have been omitted for clarity. Fragments located outside the unit cell have been completed.

## supplementary crystallographic information

### Comment

Organotrifluoroborates and, in particular, indolyltrifluoroborates [Molander *et al.* (2009), Kassis *et al.* (2009), Reiter *et al.* (2010)] are synthetically useful nucleophiles for Suzuki-Miyaura cross-coupling and other CC bond-forming reactions [Darses & Genet (2008)].

The asymmetric unit contains two formula units of the title compound (Fig. 1). The B–C bond distances in the two indolyltrifluoroborate anions are found to be 1.596 (3) Å and 1.600 (3) Å. These bond distances are close to the mean distance of 1.619 Å determined from 33 crystal structures of organotrifluoroborates (CSD version 5.33, Nov 2011). In the title compound, the 1-*tert*-butoxycarbonyl group is not coplanar with the indolyl ring, but deviates with plane-plane angles of 20.44 (11)° and 21.02 (10)°, in which the plane of a *tert*-butoxycarbonyl group is defined by its C and O atoms with the exception of the methyl groups. This structural feature is observed in several of the dozen of crystal structures of 3-substituted 1-(*tert*-Butoxycarbonyl)-1*H*-indolyl derivatives [Baran *et al.* (2005), Davies *et al.* (2005), Davies *et al.* (2007) and Lu & Lin (2011)].

The coordination sphere of K1 consists of two oxygen atoms and four fluorine atoms in bond distances ranging between 2.58 Å and 2.99 Å. Additionally the five-membered ring of an adjacent indolyl moiety is bound by a weak potassium-π interaction (distance K1–*Cg*(N2, C14—C17) = 3.722 Å). The other potassium ion is coordinated by five fluorine atoms and one oxygen atom in bond distances ranging from 2.62 Å to 2.77 Å. The water molecule is coordinated solely to K1 and forms sequential hydrogen bonds of the type O–H···O along [100]. The other proton of the water molecule acts as donor in hydrogen bonds of the type O–H···F. Layers parallel to *ab* are formed by the combination of hydrogen bonds and coordination of the potassium ions (Fig. 2). Weak C–H···π interactions between methyl-hydrogen atoms and the six-membered rings of the indolyl moieties are established with C···*Cg* distances of 3.836 (3) Å and 3.715 (2) Å. The packing of the title compound is shown in Fig. 3.

### Experimental

In a 100 ml Schlenck flask under argon, 1.00 g (2.92 mmol,1 eq.) of *tert*-butyl-3-iodo-1*H*-indole-1-carboxylate [Mothes *et al.* (2008)] was dissolved in 20 ml of freshly distilled THF and cooled to -78 °C. With a syringe, 1.37 ml of nBuLi (2.13 *M* in hexane, 2.92 mmol,1 eq.) was added dropwise, and the solution was stirred for 30 min at this temperature whilst turning orange pale. Neat triisopropyl borate (3.36 mmol, 0.55 ml, 1.15 eq.) was then slowly dropped to the mixture, and after removing the cooling bath the temperature reached 0 °C in 30 min. An aqueous solution of KHF_2_ (17.5 mmol, 1.35 g, 6 eq. dissolved in 5 ml of H_2_O) was added slowly to the limpid solution under vigorous stirring and after 15 minutes the solvents were removed *in vacuo*. The waxy solid was dissolved in 50 ml of hot acetone and filtrated. The filtrate was concentrated to 10 ml before adding 10 ml of diethylether. After one night in the fridge, colorless crystalline plates of the title compound were obtained (650 mg, 70%).

### Refinement

C-bound H atoms were positioned geometrically (C—H = 0.98 Å for aliphatic, 0.95 Å for aromatic H) and treated as riding on their parent atoms [*U*_iso_(H) = 1.2*U*_eq_(C, aromatic), *U*_iso_(H) = 1.5*U*_eq_(C, aliphatic)]. The methyl groups were allowed to rotate along the C–O bonds to best fit the experimental electron density. The hydrogen atoms of the water molecule were fixed to O–H distances of 0.82 (1) Å [*U*_iso_(H) = 1.2*U*_eq_(O)].

### Crystal data

**Table:**

K^+^·C_13_H_14_BF_3_NO_2_·0.5H_2_O	*F*(000) = 1368
*M**_r_* = 332.17	*D*_x_ = 1.441 (1) Mg m^−^^3^
Orthorhombic, *P*2_1_2_1_2_1_	Mo *K*α radiation, λ = 0.71073 Å
Hall symbol: P 2ac 2ab	Cell parameters from 12366 reflections
*a* = 5.8428 (1) Å	θ = 3.1–27.5°
*b* = 16.3177 (2) Å	µ = 0.38 mm^−^^1^
*c* = 32.1286 (5) Å	*T* = 173 K
*V* = 3063.17 (8) Å^3^	Block, colourless
*Z* = 8	0.27 × 0.19 × 0.10 mm

### Data collection

**Table:**

Nonius KappaCCD diffractometer	6369 reflections with *I* > 2σ(*I*)
Radiation source: rotating anode	*R*_int_ = 0.027
MONTEL, graded multilayered X-ray optics monochromator	θ_max_ = 27.5°, θ_min_ = 3.1°
CCD; rotation images scans	*h* = −7→7
24771 measured reflections	*k* = −21→21
7001 independent reflections	*l* = −41→41

### Refinement

**Table:**

Refinement on *F*^2^	Secondary atom site location: difference Fourier map
Least-squares matrix: full	Hydrogen site location: inferred from neighbouring sites
*R*[*F*^2^ > 2σ(*F*^2^)] = 0.032	H atoms treated by a mixture of independent and constrained refinement
*wR*(*F*^2^) = 0.075	*w* = 1/[σ^2^(*F*_o_^2^) + (0.0299*P*)^2^ + 1.0137*P*] where *P* = (*F*_o_^2^ + 2*F*_c_^2^)/3
*S* = 1.03	(Δ/σ)_max_ = 0.001
7001 reflections	Δρ_max_ = 0.30 e Å^−^^3^
400 parameters	Δρ_min_ = −0.24 e Å^−^^3^
2 restraints	Absolute structure: Flack (1983), 2995 Friedel pairs
Primary atom site location: structure-invariant direct methods	Flack parameter: 0.00 (3)

### Special details

**Table:**

Geometry. All e.s.d.'s (except the e.s.d. in the dihedral angle between two l.s. planes) are estimated using the full covariance matrix. The cell e.s.d.'s are taken into account individually in the estimation of e.s.d.'s in distances, angles and torsion angles; correlations between e.s.d.'s in cell parameters are only used when they are defined by crystal symmetry. An approximate (isotropic) treatment of cell e.s.d.'s is used for estimating e.s.d.'s involving l.s. planes.
Refinement. Refinement of *F*^2^ against ALL reflections. The weighted *R*-factor *wR* and goodness of fit *S* are based on *F*^2^, conventional *R*-factors *R* are based on *F*, with *F* set to zero for negative *F*^2^. The threshold expression of *F*^2^ > 2*σ*(*F*^2^) is used only for calculating *R*-factors(gt) *etc*. and is not relevant to the choice of reflections for refinement. *R*-factors based on *F*^2^ are statistically about twice as large as those based on *F*, and *R*-factors based on all data will be even larger.

### Fractional atomic coordinates and isotropic or equivalent isotropic displacement parameters (Å^2^)

**Table:**

	*x*	*y*	*z*	*U*_iso_*/*U*_eq_	
K1	0.23271 (7)	−0.07549 (2)	−0.010496 (14)	0.03663 (10)	
K2	−0.44437 (8)	0.16361 (2)	0.030974 (13)	0.03596 (10)	
F1	−0.1583 (2)	−0.09666 (9)	0.02545 (4)	0.0531 (3)	
F2	−0.4208 (3)	0.00302 (7)	0.02809 (4)	0.0579 (4)	
F3	−0.4763 (2)	−0.11459 (8)	0.06318 (4)	0.0515 (3)	
F4	1.2008 (3)	0.08962 (7)	−0.00590 (4)	0.0599 (4)	
F5	0.9079 (2)	0.17683 (7)	−0.01961 (4)	0.0475 (3)	
F6	1.2432 (2)	0.19073 (7)	−0.05353 (4)	0.0441 (3)	
O1	0.2775 (2)	0.13553 (7)	0.17310 (4)	0.0355 (3)	
O2	0.3463 (3)	0.14600 (10)	0.10405 (4)	0.0501 (4)	
O3	0.6469 (2)	−0.13910 (7)	−0.13800 (4)	0.0331 (3)	
O4	0.4899 (3)	−0.10246 (8)	−0.07628 (4)	0.0427 (3)	
O5	0.0801 (3)	−0.23252 (10)	−0.01815 (6)	0.0562 (4)	
H51	0.097 (5)	−0.2697 (12)	−0.0347 (7)	0.067*	
H52	−0.052 (2)	−0.2317 (18)	−0.0103 (9)	0.067*	
N1	0.0878 (3)	0.05375 (9)	0.12880 (5)	0.0323 (3)	
N2	0.7777 (3)	−0.02748 (9)	−0.10475 (4)	0.0294 (3)	
C1	0.0068 (3)	0.03130 (11)	0.08919 (6)	0.0332 (4)	
H1	0.0749	0.0484	0.0638	0.040*	
C2	−0.1791 (3)	−0.01743 (10)	0.09157 (6)	0.0289 (4)	
C3	−0.2240 (3)	−0.02728 (10)	0.13595 (5)	0.0285 (4)	
C4	−0.0545 (3)	0.01594 (10)	0.15856 (5)	0.0290 (4)	
C5	−0.0473 (4)	0.01580 (11)	0.20163 (6)	0.0362 (4)	
H5	0.0708	0.0433	0.2164	0.043*	
C6	−0.2203 (4)	−0.02636 (12)	0.22244 (6)	0.0404 (5)	
H6	−0.2196	−0.0279	0.2520	0.049*	
C7	−0.3937 (4)	−0.06622 (12)	0.20090 (6)	0.0412 (5)	
H7	−0.5118	−0.0930	0.2160	0.049*	
C8	−0.3975 (3)	−0.06758 (11)	0.15802 (6)	0.0339 (4)	
H8	−0.5162	−0.0955	0.1436	0.041*	
C9	0.2502 (4)	0.11582 (11)	0.13339 (6)	0.0343 (4)	
C10	0.4090 (4)	0.21055 (11)	0.18483 (6)	0.0359 (4)	
C11	0.6548 (4)	0.20440 (15)	0.17096 (10)	0.0607 (7)	
H11A	0.7180	0.1515	0.1796	0.091*	
H11B	0.7438	0.2488	0.1836	0.091*	
H11C	0.6625	0.2090	0.1406	0.091*	
C12	0.2916 (4)	0.28447 (12)	0.16649 (8)	0.0499 (6)	
H12A	0.3055	0.2831	0.1361	0.075*	
H12B	0.3636	0.3344	0.1772	0.075*	
H12C	0.1294	0.2839	0.1742	0.075*	
C13	0.3855 (7)	0.20916 (19)	0.23156 (8)	0.0838 (11)	
H13A	0.2229	0.2093	0.2391	0.126*	
H13B	0.4599	0.2577	0.2434	0.126*	
H13C	0.4583	0.1596	0.2426	0.126*	
C14	0.8113 (3)	0.02142 (11)	−0.06941 (6)	0.0318 (4)	
H14	0.7153	0.0204	−0.0456	0.038*	
C15	0.9966 (3)	0.07020 (10)	−0.07336 (5)	0.0275 (4)	
C16	1.0897 (3)	0.05186 (9)	−0.11437 (5)	0.0258 (3)	
C17	0.9503 (3)	−0.00726 (10)	−0.13362 (5)	0.0265 (3)	
C18	0.9858 (3)	−0.03336 (11)	−0.17426 (6)	0.0341 (4)	
H18	0.8871	−0.0719	−0.1873	0.041*	
C19	1.1730 (4)	−0.00034 (13)	−0.19486 (6)	0.0416 (5)	
H19	1.2024	−0.0166	−0.2227	0.050*	
C20	1.3184 (4)	0.05562 (12)	−0.17607 (7)	0.0412 (5)	
H20	1.4465	0.0760	−0.1911	0.049*	
C21	1.2797 (3)	0.08230 (11)	−0.13564 (6)	0.0334 (4)	
H21	1.3802	0.1204	−0.1228	0.040*	
C22	0.6229 (3)	−0.09235 (11)	−0.10457 (6)	0.0318 (4)	
C23	0.5320 (3)	−0.22094 (11)	−0.14000 (6)	0.0335 (4)	
C24	0.2743 (4)	−0.21102 (13)	−0.14087 (9)	0.0508 (6)	
H24A	0.2026	−0.2642	−0.1466	0.076*	
H24B	0.2320	−0.1720	−0.1627	0.076*	
H24C	0.2214	−0.1905	−0.1139	0.076*	
C25	0.6211 (4)	−0.25395 (13)	−0.18113 (7)	0.0471 (5)	
H25A	0.7885	−0.2570	−0.1802	0.071*	
H25B	0.5741	−0.2173	−0.2038	0.071*	
H25C	0.5581	−0.3088	−0.1860	0.071*	
C26	0.6157 (4)	−0.27308 (13)	−0.10437 (7)	0.0478 (5)	
H26A	0.7833	−0.2720	−0.1036	0.072*	
H26B	0.5631	−0.3296	−0.1082	0.072*	
H26C	0.5550	−0.2515	−0.0781	0.072*	
B1	−0.3097 (3)	−0.05677 (11)	0.05280 (6)	0.0262 (4)	
B2	1.0867 (4)	0.13229 (12)	−0.03848 (6)	0.0298 (4)	

### Atomic displacement parameters (Å^2^)

**Table:**

	*U*^11^	*U*^22^	*U*^33^	*U*^12^	*U*^13^	*U*^23^
K1	0.0355 (2)	0.03093 (19)	0.0434 (2)	−0.00376 (18)	0.00348 (19)	−0.00575 (17)
K2	0.0414 (2)	0.02571 (18)	0.0408 (2)	−0.00664 (17)	0.00910 (19)	−0.00547 (16)
F1	0.0414 (7)	0.0679 (8)	0.0500 (7)	−0.0022 (6)	0.0071 (6)	−0.0286 (6)
F2	0.0777 (9)	0.0317 (6)	0.0642 (8)	0.0021 (7)	−0.0338 (8)	−0.0013 (5)
F3	0.0583 (8)	0.0541 (7)	0.0420 (7)	−0.0296 (7)	0.0088 (6)	−0.0065 (5)
F4	0.0919 (11)	0.0359 (6)	0.0519 (7)	−0.0013 (7)	−0.0370 (8)	−0.0022 (5)
F5	0.0419 (7)	0.0433 (6)	0.0574 (8)	−0.0076 (6)	0.0157 (6)	−0.0225 (5)
F6	0.0435 (7)	0.0332 (6)	0.0558 (7)	−0.0152 (6)	0.0148 (6)	−0.0137 (5)
O1	0.0416 (8)	0.0321 (6)	0.0327 (7)	−0.0097 (6)	−0.0022 (6)	−0.0023 (5)
O2	0.0538 (10)	0.0598 (10)	0.0367 (8)	−0.0258 (8)	0.0127 (7)	−0.0080 (7)
O3	0.0378 (7)	0.0276 (6)	0.0340 (7)	−0.0098 (5)	−0.0004 (6)	−0.0035 (5)
O4	0.0422 (8)	0.0414 (8)	0.0446 (8)	−0.0145 (7)	0.0127 (7)	−0.0065 (6)
O5	0.0654 (11)	0.0346 (8)	0.0688 (12)	−0.0079 (8)	0.0163 (10)	−0.0192 (7)
N1	0.0332 (9)	0.0338 (8)	0.0298 (8)	−0.0077 (7)	0.0014 (7)	−0.0021 (6)
N2	0.0310 (8)	0.0291 (7)	0.0280 (7)	−0.0059 (6)	0.0025 (6)	−0.0019 (6)
C1	0.0350 (10)	0.0363 (9)	0.0283 (9)	−0.0063 (8)	0.0040 (7)	−0.0039 (7)
C2	0.0301 (9)	0.0234 (8)	0.0333 (9)	−0.0002 (7)	0.0028 (7)	−0.0010 (7)
C3	0.0303 (9)	0.0227 (8)	0.0325 (9)	0.0042 (7)	0.0031 (7)	0.0001 (6)
C4	0.0293 (9)	0.0264 (8)	0.0314 (9)	0.0032 (8)	0.0017 (8)	0.0022 (7)
C5	0.0416 (11)	0.0345 (9)	0.0324 (9)	0.0009 (9)	−0.0057 (9)	0.0041 (7)
C6	0.0509 (13)	0.0414 (10)	0.0290 (9)	0.0011 (10)	0.0012 (9)	0.0050 (8)
C7	0.0468 (12)	0.0350 (10)	0.0418 (11)	−0.0037 (9)	0.0098 (10)	0.0040 (8)
C8	0.0343 (10)	0.0276 (9)	0.0399 (10)	0.0001 (8)	0.0030 (8)	0.0008 (7)
C9	0.0343 (10)	0.0336 (9)	0.0350 (9)	−0.0044 (9)	0.0017 (9)	−0.0044 (7)
C10	0.0375 (11)	0.0302 (9)	0.0400 (10)	−0.0056 (8)	−0.0069 (9)	−0.0050 (8)
C11	0.0311 (11)	0.0428 (12)	0.108 (2)	0.0024 (10)	−0.0107 (13)	−0.0169 (13)
C12	0.0343 (12)	0.0331 (10)	0.0822 (17)	0.0015 (9)	−0.0019 (11)	0.0036 (10)
C13	0.135 (3)	0.0742 (19)	0.0425 (14)	−0.054 (2)	−0.0084 (17)	−0.0072 (13)
C14	0.0348 (10)	0.0327 (9)	0.0279 (9)	−0.0042 (8)	0.0026 (7)	−0.0050 (7)
C15	0.0310 (9)	0.0241 (8)	0.0274 (8)	−0.0007 (7)	0.0005 (7)	0.0006 (6)
C16	0.0274 (9)	0.0205 (7)	0.0296 (9)	0.0013 (7)	0.0011 (7)	0.0028 (6)
C17	0.0276 (8)	0.0230 (8)	0.0290 (8)	0.0011 (7)	−0.0007 (7)	0.0024 (6)
C18	0.0435 (11)	0.0300 (9)	0.0290 (9)	−0.0046 (8)	0.0016 (8)	−0.0018 (7)
C19	0.0533 (13)	0.0384 (10)	0.0331 (10)	−0.0045 (10)	0.0127 (9)	−0.0047 (8)
C20	0.0435 (11)	0.0372 (10)	0.0429 (11)	−0.0076 (9)	0.0174 (9)	−0.0015 (8)
C21	0.0319 (10)	0.0286 (8)	0.0396 (10)	−0.0046 (8)	0.0031 (8)	−0.0010 (7)
C22	0.0317 (10)	0.0298 (9)	0.0338 (9)	−0.0054 (7)	−0.0013 (8)	−0.0011 (7)
C23	0.0301 (10)	0.0268 (8)	0.0435 (10)	−0.0073 (8)	−0.0057 (8)	−0.0037 (7)
C24	0.0318 (11)	0.0388 (11)	0.0818 (17)	−0.0052 (9)	−0.0098 (11)	−0.0083 (11)
C25	0.0520 (14)	0.0404 (11)	0.0488 (13)	−0.0093 (10)	−0.0064 (11)	−0.0128 (9)
C26	0.0493 (14)	0.0346 (10)	0.0595 (14)	−0.0018 (10)	−0.0088 (11)	0.0058 (10)
B1	0.0258 (10)	0.0216 (9)	0.0313 (10)	0.0000 (7)	0.0003 (8)	−0.0012 (7)
B2	0.0339 (11)	0.0230 (9)	0.0325 (10)	−0.0025 (8)	−0.0013 (9)	−0.0011 (7)

### Geometric parameters (Å, º)

**Table:**

K1—F1	2.5832 (13)	C3—C4	1.416 (3)
K1—O4	2.6303 (14)	C4—C5	1.384 (2)
K1—F2^i^	2.6977 (13)	C5—C6	1.394 (3)
K1—F4^ii^	2.7048 (12)	C5—H5	0.9500
K1—O5	2.7243 (16)	C6—C7	1.389 (3)
K1—F3^i^	2.9833 (14)	C6—H6	0.9500
K1—B1^i^	3.373 (2)	C7—C8	1.378 (3)
K1—C15^ii^	3.4111 (17)	C7—H7	0.9500
K1—C14^ii^	3.485 (2)	C8—H8	0.9500
K1—H52	3.05 (3)	C10—C11	1.507 (3)
K2—F2	2.6256 (12)	C10—C12	1.508 (3)
K2—F5^ii^	2.6315 (12)	C10—C13	1.508 (3)
K2—O2^ii^	2.6627 (14)	C11—H11A	0.9800
K2—F4^iii^	2.6756 (14)	C11—H11B	0.9800
K2—F6^iv^	2.7158 (12)	C11—H11C	0.9800
K2—F5^iv^	2.7671 (12)	C12—H12A	0.9800
K2—F6^iii^	3.3016 (14)	C12—H12B	0.9800
K2—B2^iv^	3.344 (2)	C12—H12C	0.9800
F1—B1	1.406 (2)	C13—H13A	0.9800
F2—B1	1.415 (2)	C13—H13B	0.9800
F2—K1^ii^	2.6977 (13)	C13—H13C	0.9800
F3—B1	1.396 (2)	C14—C15	1.350 (2)
F3—K1^ii^	2.9832 (14)	C14—K1^i^	3.485 (2)
F4—B2	1.423 (2)	C14—H14	0.9500
F4—K2^v^	2.6756 (14)	C15—C16	1.457 (2)
F4—K1^i^	2.7047 (12)	C15—B2	1.600 (3)
F5—B2	1.409 (2)	C15—K1^i^	3.4111 (17)
F5—K2^i^	2.6316 (12)	C16—C21	1.395 (2)
F5—K2^vi^	2.7671 (12)	C16—C17	1.406 (2)
F6—B2	1.407 (2)	C17—C18	1.389 (2)
F6—K2^vi^	2.7157 (12)	C18—C19	1.387 (3)
F6—K2^v^	3.3017 (14)	C18—H18	0.9500
O1—C9	1.325 (2)	C19—C20	1.386 (3)
O1—C10	1.494 (2)	C19—H19	0.9500
O2—C9	1.203 (2)	C20—C21	1.388 (3)
O2—K2^i^	2.6627 (14)	C20—H20	0.9500
O3—C22	1.325 (2)	C21—H21	0.9500
O3—C23	1.496 (2)	C23—C26	1.508 (3)
O4—C22	1.207 (2)	C23—C24	1.515 (3)
O5—H51	0.812 (10)	C23—C25	1.519 (3)
O5—H52	0.813 (10)	C24—H24A	0.9800
N1—C9	1.396 (2)	C24—H24B	0.9800
N1—C1	1.406 (2)	C24—H24C	0.9800
N1—C4	1.409 (2)	C25—H25A	0.9800
N2—C22	1.393 (2)	C25—H25B	0.9800
N2—C14	1.402 (2)	C25—H25C	0.9800
N2—C17	1.409 (2)	C26—H26A	0.9800
C1—C2	1.348 (3)	C26—H26B	0.9800
C1—H1	0.9500	C26—H26C	0.9800
C2—C3	1.459 (2)	B1—K1^ii^	3.373 (2)
C2—B1	1.596 (3)	B2—K2^vi^	3.344 (2)
C3—C8	1.401 (3)		
			
F1—K1—O4	147.37 (4)	K1—O5—H52	106 (2)
F1—K1—F2^i^	121.45 (5)	H51—O5—H52	109 (3)
O4—K1—F2^i^	91.15 (5)	C9—N1—C1	120.89 (15)
F1—K1—F4^ii^	92.74 (5)	C9—N1—C4	130.33 (15)
O4—K1—F4^ii^	104.47 (5)	C1—N1—C4	107.54 (15)
F2^i^—K1—F4^ii^	63.48 (4)	C22—N2—C14	121.35 (15)
F1—K1—O5	67.98 (5)	C22—N2—C17	130.21 (15)
O4—K1—O5	87.55 (5)	C14—N2—C17	107.45 (14)
F2^i^—K1—O5	137.21 (5)	C2—C1—N1	111.93 (16)
F4^ii^—K1—O5	156.82 (6)	C2—C1—H1	124.0
F1—K1—F3^i^	96.93 (4)	N1—C1—H1	124.0
O4—K1—F3^i^	106.04 (4)	C1—C2—C3	105.39 (16)
F2^i^—K1—F3^i^	46.31 (3)	C1—C2—B1	125.33 (16)
F4^ii^—K1—F3^i^	102.06 (4)	C3—C2—B1	129.24 (16)
O5—K1—F3^i^	93.27 (5)	C8—C3—C4	118.68 (16)
F1—K1—B1^i^	116.33 (5)	C8—C3—C2	132.59 (17)
O4—K1—B1^i^	92.69 (5)	C4—C3—C2	108.70 (15)
F2^i^—K1—B1^i^	23.80 (4)	C5—C4—N1	131.36 (18)
F4^ii^—K1—B1^i^	86.07 (4)	C5—C4—C3	122.22 (17)
O5—K1—B1^i^	113.52 (6)	N1—C4—C3	106.41 (15)
F3^i^—K1—B1^i^	24.40 (4)	C4—C5—C6	117.27 (19)
F1—K1—C15^ii^	90.01 (5)	C4—C5—H5	121.4
O4—K1—C15^ii^	82.64 (5)	C6—C5—H5	121.4
F2^i^—K1—C15^ii^	104.16 (4)	C7—C6—C5	121.43 (18)
F4^ii^—K1—C15^ii^	46.38 (4)	C7—C6—H6	119.3
O5—K1—C15^ii^	118.01 (5)	C5—C6—H6	119.3
F3^i^—K1—C15^ii^	148.14 (4)	C8—C7—C6	121.14 (19)
B1^i^—K1—C15^ii^	127.92 (4)	C8—C7—H7	119.4
F1—K1—C14^ii^	71.25 (5)	C6—C7—H7	119.4
O4—K1—C14^ii^	92.47 (5)	C7—C8—C3	119.15 (19)
F2^i^—K1—C14^ii^	124.35 (4)	C7—C8—H8	120.4
F4^ii^—K1—C14^ii^	61.90 (4)	C3—C8—H8	120.4
O5—K1—C14^ii^	98.42 (5)	O2—C9—O1	126.80 (18)
F3^i^—K1—C14^ii^	158.52 (4)	O2—C9—N1	122.11 (17)
B1^i^—K1—C14^ii^	147.81 (4)	O1—C9—N1	111.08 (16)
C15^ii^—K1—C14^ii^	22.54 (4)	O1—C10—C11	111.15 (16)
F1—K1—K2^i^	110.59 (3)	O1—C10—C12	108.85 (16)
O4—K1—K2^i^	98.19 (3)	C11—C10—C12	111.8 (2)
F2^i^—K1—K2^i^	31.13 (3)	O1—C10—C13	101.06 (17)
F4^ii^—K1—K2^i^	32.38 (3)	C11—C10—C13	112.4 (2)
O5—K1—K2^i^	166.44 (5)	C12—C10—C13	111.1 (2)
F3^i^—K1—K2^i^	73.36 (2)	C10—C11—H11A	109.5
B1^i^—K1—K2^i^	54.20 (3)	C10—C11—H11B	109.5
C15^ii^—K1—K2^i^	75.08 (3)	H11A—C11—H11B	109.5
C14^ii^—K1—K2^i^	93.62 (3)	C10—C11—H11C	109.5
F1—K1—H52	53.4 (3)	H11A—C11—H11C	109.5
O4—K1—H52	100.0 (4)	H11B—C11—H11C	109.5
F2^i^—K1—H52	143.8 (5)	C10—C12—H12A	109.5
F4^ii^—K1—H52	142.8 (3)	C10—C12—H12B	109.5
O5—K1—H52	14.9 (3)	H12A—C12—H12B	109.5
F3^i^—K1—H52	97.5 (5)	C10—C12—H12C	109.5
B1^i^—K1—H52	120.5 (5)	H12A—C12—H12C	109.5
C15^ii^—K1—H52	111.3 (5)	H12B—C12—H12C	109.5
C14^ii^—K1—H52	89.7 (5)	C10—C13—H13A	109.5
K2^i^—K1—H52	161.3 (5)	C10—C13—H13B	109.5
F2—K2—F5^ii^	91.09 (5)	H13A—C13—H13B	109.5
F2—K2—O2^ii^	86.99 (5)	C10—C13—H13C	109.5
F5^ii^—K2—O2^ii^	155.88 (5)	H13A—C13—H13C	109.5
F2—K2—F4^iii^	64.83 (4)	H13B—C13—H13C	109.5
F5^ii^—K2—F4^iii^	111.69 (5)	C15—C14—N2	111.82 (16)
O2^ii^—K2—F4^iii^	89.19 (5)	C15—C14—K1^i^	75.65 (11)
F2—K2—F6^iv^	149.50 (4)	N2—C14—K1^i^	106.29 (11)
F5^ii^—K2—F6^iv^	77.14 (4)	C15—C14—H14	124.1
O2^ii^—K2—F6^iv^	92.55 (4)	N2—C14—H14	124.1
F4^iii^—K2—F6^iv^	145.66 (4)	K1^i^—C14—H14	88.3
F2—K2—F5^iv^	161.94 (4)	C14—C15—C16	105.29 (15)
F5^ii^—K2—F5^iv^	94.90 (3)	C14—C15—B2	124.85 (16)
O2^ii^—K2—F5^iv^	94.27 (5)	C16—C15—B2	129.85 (15)
F4^iii^—K2—F5^iv^	97.15 (4)	C14—C15—K1^i^	81.81 (11)
F6^iv^—K2—F5^iv^	48.52 (3)	C16—C15—K1^i^	103.96 (10)
F2—K2—F6^iii^	97.69 (3)	B2—C15—K1^i^	83.88 (10)
F5^ii^—K2—F6^iii^	85.04 (4)	C21—C16—C17	119.33 (16)
O2^ii^—K2—F6^iii^	119.05 (4)	C21—C16—C15	131.86 (16)
F4^iii^—K2—F6^iii^	42.94 (3)	C17—C16—C15	108.80 (15)
F6^iv^—K2—F6^iii^	108.98 (2)	C18—C17—C16	122.48 (16)
F5^iv^—K2—F6^iii^	65.98 (3)	C18—C17—N2	130.85 (16)
F2—K2—B2^iv^	173.53 (5)	C16—C17—N2	106.60 (14)
F5^ii^—K2—B2^iv^	85.44 (4)	C19—C18—C17	116.58 (18)
O2^ii^—K2—B2^iv^	93.94 (5)	C19—C18—H18	121.7
F4^iii^—K2—B2^iv^	121.56 (5)	C17—C18—H18	121.7
F6^iv^—K2—B2^iv^	24.11 (4)	C20—C19—C18	122.10 (18)
F5^iv^—K2—B2^iv^	24.41 (4)	C20—C19—H19	119.0
F6^iii^—K2—B2^iv^	87.47 (4)	C18—C19—H19	118.9
F2—K2—B2^iii^	82.84 (4)	C19—C20—C21	120.94 (18)
F5^ii^—K2—B2^iii^	103.06 (5)	C19—C20—H20	119.5
O2^ii^—K2—B2^iii^	100.55 (5)	C21—C20—H20	119.5
F4^iii^—K2—B2^iii^	20.62 (4)	C20—C21—C16	118.46 (17)
F6^iv^—K2—B2^iii^	127.00 (4)	C20—C21—H21	120.8
F5^iv^—K2—B2^iii^	79.22 (4)	C16—C21—H21	120.8
F6^iii^—K2—B2^iii^	23.21 (4)	O4—C22—O3	126.87 (17)
B2^iv^—K2—B2^iii^	103.25 (5)	O4—C22—N2	121.69 (17)
F2—K2—K2^vii^	129.84 (3)	O3—C22—N2	111.43 (15)
F5^ii^—K2—K2^vii^	100.51 (3)	O3—C23—C26	109.01 (15)
O2^ii^—K2—K2^vii^	99.12 (4)	O3—C23—C24	110.57 (16)
F4^iii^—K2—K2^vii^	65.53 (3)	C26—C23—C24	113.36 (19)
F6^iv^—K2—K2^vii^	80.36 (3)	O3—C23—C25	101.55 (15)
F5^iv^—K2—K2^vii^	32.19 (2)	C26—C23—C25	110.46 (18)
F6^iii^—K2—K2^vii^	36.60 (2)	C24—C23—C25	111.24 (19)
B2^iv^—K2—K2^vii^	56.35 (4)	C23—C24—H24A	109.5
B2^iii^—K2—K2^vii^	47.03 (3)	C23—C24—H24B	109.5
F2—K2—K2^viii^	124.95 (4)	H24A—C24—H24B	109.5
F5^ii^—K2—K2^viii^	34.07 (2)	C23—C24—H24C	109.5
O2^ii^—K2—K2^viii^	138.78 (3)	H24A—C24—H24C	109.5
F4^iii^—K2—K2^viii^	125.95 (3)	H24B—C24—H24C	109.5
F6^iv^—K2—K2^viii^	46.46 (3)	C23—C25—H25A	109.5
F5^iv^—K2—K2^viii^	63.69 (3)	C23—C25—H25B	109.5
F6^iii^—K2—K2^viii^	84.93 (2)	H25A—C25—H25B	109.5
B2^iv^—K2—K2^viii^	51.37 (4)	C23—C25—H25C	109.5
B2^iii^—K2—K2^viii^	108.06 (4)	H25A—C25—H25C	109.5
K2^vii^—K2—K2^viii^	80.496 (13)	H25B—C25—H25C	109.5
F2—K2—K1^ii^	32.08 (3)	C23—C26—H26A	109.5
F5^ii^—K2—K1^ii^	102.39 (3)	C23—C26—H26B	109.5
O2^ii^—K2—K1^ii^	88.57 (3)	H26A—C26—H26B	109.5
F4^iii^—K2—K1^ii^	32.77 (3)	C23—C26—H26C	109.5
F6^iv^—K2—K1^ii^	178.10 (3)	H26A—C26—H26C	109.5
F5^iv^—K2—K1^ii^	129.88 (3)	H26B—C26—H26C	109.5
F6^iii^—K2—K1^ii^	69.12 (2)	F3—B1—F1	105.97 (14)
B2^iv^—K2—K1^ii^	154.26 (4)	F3—B1—F2	106.27 (15)
B2^iii^—K2—K1^ii^	51.23 (3)	F1—B1—F2	104.90 (16)
K2^vii^—K2—K1^ii^	97.952 (11)	F3—B1—C2	114.77 (15)
K2^viii^—K2—K1^ii^	132.537 (15)	F1—B1—C2	111.89 (15)
B1—F1—K1	140.70 (11)	F2—B1—C2	112.31 (14)
B1—F2—K2	133.82 (11)	F3—B1—K1^ii^	61.98 (9)
B1—F2—K1^ii^	105.92 (10)	F1—B1—K1^ii^	94.60 (10)
K2—F2—K1^ii^	116.79 (5)	F2—B1—K1^ii^	50.28 (8)
B1—F3—K1^ii^	93.63 (10)	C2—B1—K1^ii^	152.40 (12)
B2—F4—K2^v^	117.91 (11)	F6—B2—F5	106.25 (14)
B2—F4—K1^i^	118.65 (10)	F6—B2—F4	106.25 (16)
K2^v^—F4—K1^i^	114.86 (5)	F5—B2—F4	106.46 (16)
B2—F5—K2^i^	143.41 (11)	F6—B2—C15	113.72 (15)
B2—F5—K2^vi^	101.39 (10)	F5—B2—C15	112.58 (16)
K2^i^—F5—K2^vi^	113.74 (4)	F4—B2—C15	111.08 (14)
B2—F6—K2^vi^	103.84 (10)	F6—B2—K2^vi^	52.05 (8)
B2—F6—K2^v^	89.19 (10)	F5—B2—K2^vi^	54.21 (8)
K2^vi^—F6—K2^v^	96.95 (3)	F4—B2—K2^vi^	117.36 (11)
C9—O1—C10	120.25 (14)	C15—B2—K2^vi^	131.55 (12)
C9—O2—K2^i^	161.75 (14)	F6—B2—K2^v^	67.61 (10)
C22—O3—C23	120.08 (14)	F5—B2—K2^v^	103.06 (11)
C22—O4—K1	161.78 (13)	C15—B2—K2^v^	141.36 (12)
K1—O5—H51	136 (2)	K2^vi^—B2—K2^v^	81.60 (4)
			
O4—K1—F1—B1	−147.17 (17)	C21—C16—C17—N2	178.67 (15)
F2^i^—K1—F1—B1	35.8 (2)	C15—C16—C17—N2	−1.82 (18)
F4^ii^—K1—F1—B1	−24.58 (19)	C22—N2—C17—C18	16.4 (3)
O5—K1—F1—B1	168.7 (2)	C14—N2—C17—C18	−175.10 (19)
F3^i^—K1—F1—B1	77.93 (19)	C22—N2—C17—C16	−166.55 (17)
B1^i^—K1—F1—B1	62.39 (19)	C14—N2—C17—C16	1.93 (18)
C15^ii^—K1—F1—B1	−70.90 (19)	C16—C17—C18—C19	2.4 (3)
C14^ii^—K1—F1—B1	−83.63 (19)	N2—C17—C18—C19	178.99 (18)
K2^i^—K1—F1—B1	3.15 (19)	C17—C18—C19—C20	0.2 (3)
F5^ii^—K2—F2—B1	92.47 (17)	C18—C19—C20—C21	−1.2 (3)
O2^ii^—K2—F2—B1	−63.47 (17)	C19—C20—C21—C16	−0.4 (3)
F4^iii^—K2—F2—B1	−153.99 (18)	C17—C16—C21—C20	2.9 (3)
F6^iv^—K2—F2—B1	26.4 (2)	C15—C16—C21—C20	−176.48 (19)
F5^iv^—K2—F2—B1	−158.03 (15)	K1—O4—C22—O3	−162.5 (3)
F6^iii^—K2—F2—B1	177.62 (17)	K1—O4—C22—N2	18.9 (6)
B2^iii^—K2—F2—B1	−164.50 (18)	C23—O3—C22—O4	−11.1 (3)
K2^vii^—K2—F2—B1	−162.85 (15)	C23—O3—C22—N2	167.62 (15)
K2^viii^—K2—F2—B1	88.44 (17)	C14—N2—C22—O4	10.1 (3)
K1^ii^—K2—F2—B1	−155.6 (2)	C17—N2—C22—O4	177.22 (18)
F5^ii^—K2—F2—K1^ii^	−111.94 (6)	C14—N2—C22—O3	−168.73 (16)
O2^ii^—K2—F2—K1^ii^	92.12 (7)	C17—N2—C22—O3	−1.6 (3)
F4^iii^—K2—F2—K1^ii^	1.60 (6)	C22—O3—C23—C26	−59.2 (2)
F6^iv^—K2—F2—K1^ii^	−177.97 (6)	C22—O3—C23—C24	66.1 (2)
F5^iv^—K2—F2—K1^ii^	−2.4 (2)	C22—O3—C23—C25	−175.75 (16)
F6^iii^—K2—F2—K1^ii^	−26.80 (7)	K1^ii^—F3—B1—F1	−86.68 (13)
B2^iii^—K2—F2—K1^ii^	−8.92 (6)	K1^ii^—F3—B1—F2	24.56 (13)
K2^vii^—K2—F2—K1^ii^	−7.26 (9)	K1^ii^—F3—B1—C2	149.32 (13)
K2^viii^—K2—F2—K1^ii^	−115.98 (5)	K1—F1—B1—F3	−170.68 (12)
F1—K1—O4—C22	137.5 (4)	K1—F1—B1—F2	77.1 (2)
F2^i^—K1—O4—C22	−45.0 (4)	K1—F1—B1—C2	−44.9 (2)
F4^ii^—K1—O4—C22	17.9 (4)	K1—F1—B1—K1^ii^	127.17 (15)
O5—K1—O4—C22	177.8 (4)	K2—F2—B1—F3	128.95 (14)
F3^i^—K1—O4—C22	−89.5 (4)	K1^ii^—F2—B1—F3	−28.49 (15)
B1^i^—K1—O4—C22	−68.7 (4)	K2—F2—B1—F1	−119.07 (15)
C15^ii^—K1—O4—C22	59.1 (4)	K1^ii^—F2—B1—F1	83.50 (13)
C14^ii^—K1—O4—C22	79.5 (4)	K2—F2—B1—C2	2.7 (2)
K2^i^—K1—O4—C22	−14.5 (4)	K1^ii^—F2—B1—C2	−154.75 (12)
C9—N1—C1—C2	−167.77 (17)	K2—F2—B1—K1^ii^	157.44 (19)
C4—N1—C1—C2	0.8 (2)	C1—C2—B1—F3	170.96 (17)
N1—C1—C2—C3	0.3 (2)	C3—C2—B1—F3	−6.6 (3)
N1—C1—C2—B1	−177.75 (16)	C1—C2—B1—F1	50.2 (2)
C1—C2—C3—C8	176.85 (19)	C3—C2—B1—F1	−127.44 (19)
B1—C2—C3—C8	−5.2 (3)	C1—C2—B1—F2	−67.5 (2)
C1—C2—C3—C4	−1.3 (2)	C3—C2—B1—F2	114.9 (2)
B1—C2—C3—C4	176.68 (16)	C1—C2—B1—K1^ii^	−112.6 (3)
C9—N1—C4—C5	−14.9 (3)	C3—C2—B1—K1^ii^	69.8 (3)
C1—N1—C4—C5	178.0 (2)	K2^vi^—F6—B2—F5	0.78 (16)
C9—N1—C4—C3	165.54 (18)	K2^v^—F6—B2—F5	97.73 (13)
C1—N1—C4—C3	−1.53 (19)	K2^vi^—F6—B2—F4	−112.32 (13)
C8—C3—C4—C5	3.7 (3)	K2^v^—F6—B2—F4	−15.37 (13)
C2—C3—C4—C5	−177.83 (17)	K2^vi^—F6—B2—C15	125.18 (13)
C8—C3—C4—N1	−176.70 (15)	K2^v^—F6—B2—C15	−137.87 (14)
C2—C3—C4—N1	1.74 (19)	K2^v^—F6—B2—K2^vi^	96.95 (5)
N1—C4—C5—C6	178.10 (18)	K2^vi^—F6—B2—K2^v^	−96.95 (5)
C3—C4—C5—C6	−2.5 (3)	K2^i^—F5—B2—F6	−164.53 (12)
C4—C5—C6—C7	−0.4 (3)	K2^vi^—F5—B2—F6	−0.76 (15)
C5—C6—C7—C8	1.9 (3)	K2^i^—F5—B2—F4	−51.6 (2)
C6—C7—C8—C3	−0.6 (3)	K2^vi^—F5—B2—F4	112.20 (12)
C4—C3—C8—C7	−2.1 (3)	K2^i^—F5—B2—C15	70.4 (2)
C2—C3—C8—C7	179.89 (19)	K2^vi^—F5—B2—C15	−125.86 (12)
K2^i^—O2—C9—O1	142.3 (4)	K2^i^—F5—B2—K2^vi^	−163.8 (2)
K2^i^—O2—C9—N1	−38.6 (6)	K2^i^—F5—B2—K2^v^	−94.39 (17)
C10—O1—C9—O2	10.7 (3)	K2^vi^—F5—B2—K2^v^	69.38 (8)
C10—O1—C9—N1	−168.51 (15)	K2^v^—F4—B2—F6	21.72 (18)
C1—N1—C9—O2	−8.6 (3)	K1^i^—F4—B2—F6	−124.53 (12)
C4—N1—C9—O2	−174.2 (2)	K2^v^—F4—B2—F5	−91.24 (15)
C1—N1—C9—O1	170.70 (17)	K1^i^—F4—B2—F5	122.51 (12)
C4—N1—C9—O1	5.1 (3)	K2^v^—F4—B2—C15	145.88 (12)
C9—O1—C10—C11	−62.8 (2)	K1^i^—F4—B2—C15	−0.4 (2)
C9—O1—C10—C12	60.7 (2)	K2^v^—F4—B2—K2^vi^	−33.50 (17)
C9—O1—C10—C13	177.7 (2)	K1^i^—F4—B2—K2^vi^	−179.74 (6)
C22—N2—C14—C15	168.35 (16)	K1^i^—F4—B2—K2^v^	−146.25 (17)
C17—N2—C14—C15	−1.4 (2)	C14—C15—B2—F6	−164.37 (17)
C22—N2—C14—K1^i^	87.62 (16)	C16—C15—B2—F6	17.0 (3)
C17—N2—C14—K1^i^	−82.10 (13)	K1^i^—C15—B2—F6	120.06 (14)
N2—C14—C15—C16	0.2 (2)	C14—C15—B2—F5	−43.5 (2)
K1^i^—C14—C15—C16	102.32 (12)	C16—C15—B2—F5	137.88 (18)
N2—C14—C15—B2	−178.71 (16)	K1^i^—C15—B2—F5	−119.03 (14)
K1^i^—C14—C15—B2	−76.62 (16)	C14—C15—B2—F4	75.8 (2)
N2—C14—C15—K1^i^	−102.09 (14)	C16—C15—B2—F4	−102.8 (2)
C14—C15—C16—C21	−179.56 (18)	K1^i^—C15—B2—F4	0.26 (15)
B2—C15—C16—C21	−0.7 (3)	C14—C15—B2—K2^vi^	−104.92 (19)
K1^i^—C15—C16—C21	−94.39 (19)	C16—C15—B2—K2^vi^	76.4 (2)
C14—C15—C16—C17	1.01 (19)	K1^i^—C15—B2—K2^vi^	179.51 (13)
B2—C15—C16—C17	179.87 (17)	C14—C15—B2—K2^v^	112.3 (2)
K1^i^—C15—C16—C17	86.18 (13)	C16—C15—B2—K2^v^	−66.3 (3)
C21—C16—C17—C18	−4.0 (3)	K1^i^—C15—B2—K2^v^	36.76 (17)
C15—C16—C17—C18	175.51 (16)		

Symmetry codes: (i) *x*+1, *y*, *z*; (ii) *x*−1, *y*, *z*; (iii) *x*−2, *y*, *z*; (iv) *x*−3/2, −*y*+1/2, −*z*; (v) *x*+2, *y*, *z*; (vi) *x*+3/2, −*y*+1/2, −*z*; (vii) *x*−1/2, −*y*+1/2, −*z*; (viii) *x*+1/2, −*y*+1/2, −*z*.

### Hydrogen-bond geometry (Å, º)

**Table:**

*D*—H···*A*	*D*—H	H···*A*	*D*···*A*	*D*—H···*A*
O5—H51···F3^ix^	0.81 (1)	2.14 (1)	2.903 (2)	156 (3)
O5—H51···F1^ix^	0.81 (1)	2.62 (2)	3.188 (2)	128 (2)
O5—H52···O5^x^	0.81 (1)	2.41 (1)	3.1968 (16)	164 (3)

Symmetry codes: (ix) *x*+1/2, −*y*−1/2, −*z*; (x) *x*−1/2, −*y*−1/2, −*z*.
